# Network controllability solutions for computational drug repurposing using genetic algorithms

**DOI:** 10.1038/s41598-022-05335-3

**Published:** 2022-01-26

**Authors:** Victor-Bogdan Popescu, Krishna Kanhaiya, Dumitru Iulian Năstac, Eugen Czeizler, Ion Petre

**Affiliations:** 1 Computer Science, Åbo Akademi University, 20500 Turku, Finland; 2 POLITEHNICA University of Bucharest, Faculty of Electronics, Telecommunications and Information Technology, 061071 Bucharest, Romania; 3 Department of Mathematics and Statistics, University of Turku, 20014 Turku, Finland; 4 National Institute for Research and Development in Biological Sciences, 060031 Bucharest, Romania

**Keywords:** Oncology, Network topology, Control theory, Combination drug therapy

## Abstract

Control theory has seen recently impactful applications in network science, especially in connections with applications in network medicine. A key topic of research is that of finding minimal external interventions that offer control over the dynamics of a given network, a problem known as network controllability. We propose in this article a new solution for this problem based on genetic algorithms. We tailor our solution for applications in computational drug repurposing, seeking to maximize its use of FDA-approved drug targets in a given disease-specific protein-protein interaction network. We demonstrate our algorithm on several cancer networks and on several random networks with their edges distributed according to the Erdős–Rényi, the Scale-Free, and the Small World properties. Overall, we show that our new algorithm is more efficient in identifying relevant drug targets in a disease network, advancing the computational solutions needed for new therapeutic and drug repurposing approaches.

## Introduction

Network modeling in systems medicine has emerged as a powerful analytics approach in the last couple of decades^1,2^. Its aim is to analyze diseases and drug interventions as ways of acting over bio-medical dynamical networks^3,4^, such as protein-protein interaction networks^5,6^, signalling networks^7^, metabolic networks^8^, and immunological responses^9^. In this framework, a disease is seen as emerging from some of its modules being affected (directly or through cascading signals) and from critical nodes in the network being deregulated^10^. Similarly, drug therapies are seen as outside controlled interventions within a deregulated network with the aim of either re-balancing the system or possibly isolating some specific components of the network^11^. A particular advantage of this approach is reasoning about multiple-drug interventions, analyzing and predicting multi-drug synergies. Instead of acting over each individual dysregulated component, one can try to influence several of these entities through a few well-chosen interventions, and to have them spread in cascade into the network using the network’s own internal connections. Network controllability is a topic of high relevance in this area with a rich theory to support it^12^. It has found in recent years powerful applications in computational systems medicine and therapeutics^5,6,13,14^.

The theory of network controllability aims at providing a sound and theoretically accurate description of what control means within a network, and how it can be achieved. Intuitively, achieving control over a system from a set of input nodes means being able to drive that system from any initial setup to any desired state. This is an intrinsic global optimization problem with the objective to minimize the number of input nodes needed for the control. Additional constraints may be added depending on the application, such as the control pathways from the input nodes to the controlled nodes to be short, or the input nodes to be primarily selected from a given set of preferred nodes (e.g., targets of standard therapy drugs). This leads to several problem variations, such as: structural controllability^13^ (identifying pathways that offer control over the system regardless of its numerical setup); target controllability^14^ (achieving control over a predefined set of target nodes); constrained target controllability^15^ (selecting the input nodes from a pre-defined preferred set); target controllability with minimal mediators^16^ (avoiding specific nodes that could cause side-effects); minimum dominating sets^17^ (finding a minimal set of nodes that are one step upstream of all other nodes in the network). Some of these optimization problems are known to have efficient algorithmic solutions^13^. Others, on the contrary, are known to be computationally difficult, yet approximate efficient solutions are still achievable^5^. Recent successful applications of network controllability include research on the contribution of individual neurons in the locomotion of *C. elegans*^18^, and on the discovery of potential drug combinations for leukemia^19^, breast cancer and COVID-19^20^.

Motivated by the applicability of network control in systems medicine, the problem we focus on in this paper is minimizing the number of external interventions needed to achieve structural target control of a system. We are particularly interested in the case where the targets are disease-specific survivability-essential genes, key targets for synthetic lethality^21^. We identify control interventions that are achievable through the delivery of FDA-approved drugs, by giving a preference to FDA-approved drug targets being selected as input nodes. The minimization of the number of input nodes is part of the standard optimization objective of the network controllability problem and there is an additional incentive to minimize them in the medical case studies: combinatorial drug therapies can only include few simultaneously delivered drugs. The optimization goal of the network controllability problem can address this constraint by selecting the input nodes among the drug-approved nodes.

**Figure 1:**
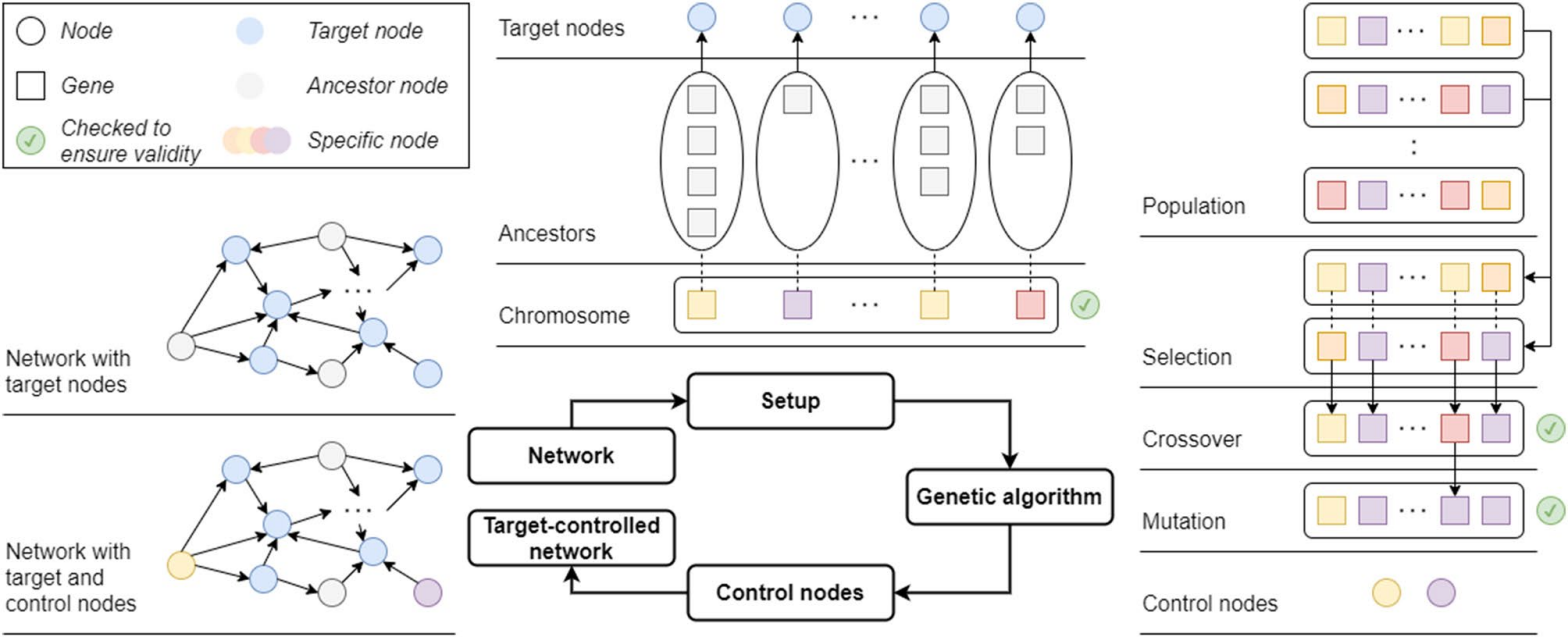
The basic outline of the genetic algorithm, shown in a clockwise manner: the required data, the initial setup and chromosome encoding, the operators of the genetic algorithm, and the chromosome decoding and final result. In brief, the algorithm starts with an initialization stage, where it generates a “population” of control sets for the target set; the controllability is verified through the Kalman rank condition^12^. It then attempts to generate control sets of smaller and smaller size through combinations of the current control sets (crossover and mutation) and through adding new control sets to the population of solutions.

The structural target controllability problem is known to be NP-hard^5^, meaning that finding the smallest set of inputs for controlling the target set is computationally prohibitive for large networks. Several greedy-based approximations of the optimal solutions have been proposed for different variants of the problem^5,14–16^, also recent solutions based on linear integer programming^20^. In our experience^6,22^, the greedy algorithm tends to select few preferred input nodes in each solution. This is understandable since its search is based on consecutive edge selections that may lead it away from the preferred input nodes. To address this problem, we propose a new solution based on genetic algorithms, a well known heuristic choice for nonlinear optimization problems^23^. This offers a different approach to searching for a solution to network controllability: the search is on suitable combinations of input nodes that control the set of target nodes. We maximize the use of the preferred input nodes in each step of the algorithm, to get a considerably larger selection of such nodes in the solution. An overview of the basic outline of the genetic algorithm is presented in Fig. 1 and discussed in details in the “Methods” section.

Drug repurposing, i.e., identifying novel uses for already existing drugs, has received significant attention due to its potential for efficient advances in drug development, leading to a significantly shorter timeline and reduced costs^24^. In addition to the standard experimental approach, recent advances in computational methods and the availability of high-quality big data enabled the evolution of efficient and promising computational approaches. Moreover, the urgency of the current COVID-19 pandemic has brought additional interest in the potential of computational drug repurposing^22,25,26^. Multiple computational methods have been successfully applied to the identification of drug repurposing candidates: machine learning (neural networks^27^, deep learning^28^, support vector machines and random forests^29^), data mining (text mining^30^, semantic inference^31^), and network analysis (clustering^32^, centrality measures^33^, controllability^26^). Our method can be placed at the intersection of AI- and network-based computational drug repurposing approaches, using evolutionary algorithms rooted in the structure of interaction networks and integrating additional disease- and drug-data.

## Results

We tested the genetic algorithm on 15 random directed networks, generated using the NetworkX Python package^34^, with the number of nodes ranging from 100 to 2000 and the edges distributed according to the Erdős–Rényi-, the Scale-Free-, and the Small World-graph edge distributions. For each network, the target sets consisted of $5\%$ randomly selected nodes with positive in-degree (which gives them a chance to be controlled). Furthermore, as a proof-of-concept, we applied the algorithm on 9 breast, pancreatic, and ovarian cancer cell line-specific directed protein–protein interaction networks^6,35^. These networks were constructed based on protein data from the UniProtKB database^36^ and interaction data from the SIGNOR database^37^. We used as control targets the cancer-essential genes specific to each cell line^35^, and as preferred control inputs the FDA-approved drug-targets of DrugBank^38^. An overview of the networks is presented in Table 1 (the number of nodes, edges, preferred nodes, and target nodes), in Supplementary Fig. S1 (the out-degree centrality, the closeness centrality, and the betweenness centrality), and in Supplementary Table 1.

**Table 1:** The analyzed data sets. All isolated nodes were removed from the networks.

Type	Network	N	E	T	P	TP
Random	Erdős–Rényi 100*	62	47	5		
	Erdős–Rényi 500*	497	1270	25		
	Erdős–Rényi 1000*	1000	4952	50		
	Erdős–Rényi 1500*	1500	11258	75		
	Erdős–Rényi 2000*	2000	19869	100		
	Scale-Free 100**	100	166	5		
	Scale-Free 500**	500	816	25		
	Scale-Free 1000**	1000	1793	50		
	Scale-Free 1500**	1500	2526	75		
	Scale-Free 2000**	2000	3401	100		
	Small World 100***	100	400	5		
	Small World 500***	500	2000	25		
	Small World 1000***	1000	4000	50		
	Small World 1500***	1500	6000	75		
	Small World 2000***	2000	8000	100		
Biological	Breast DEF^6^	1415	2435	112	123	15
	Breast HCC1428^35^	1495	2650	126	135	15
	Breast MDA-MB-361^35^	1478	2590	124	136	15
	Ovarian DEF^6^	1047	1579	140	100	9
	Ovarian O1946^35^	1155	1823	159	104	10
	Ovarian OVCA8^35^	1157	1781	161	105	17
	Pancreatic AsPC-1^35^	1022	1534	125	90	7
	Pancreatic DEF^6^	991	1484	168	86	16
	Pancreatic KP-3^35^	1134	1757	167	94	12

*N* Number of nodes in the network, *E* number of edges in the network, *T* number of control target nodes in the network, *P* number of preferred (i.e., drug-targetable) nodes in the network, *TP* number of control target nodes in the network that are also preferred (i.e., drug-targetable) nodes.

*,**,***Generated using “networkx”^34^, *: “fast_gnp_random_graph” with $p = 0.005$), **: “scale_free_graph” with the default parameters, ***: “watts_strogatz_graph” with $k = 4$ and $p = 0.2$.

The approach we took in this study is based on unlabelled directed networks, i.e., where we only include the information that a certain directed interaction between two proteins exists, without the details on the nature (e.g., inhibiting or activating) or the strength of that interaction. This is indeed the most common approach to network controllability. Skipping the labelling of the interactions may lead to some false positive results regarding the therapeutic effects: some of the control pathways we identify may have a weak result due to the conflicting contributions of the interactions it consists of. A modest amount of false positives is sometimes tolerated in medical research, more than false negatives, because it leads to a wider pool of candidates to be verified experimentally. Approaches representing the type of interaction within the network and within the controllability problem do exist, e.g., based on Boolean networks^39,40^, but they are hindered by major scalability problems.

We compared the results of the genetic algorithm to the results of the greedy algorithm for structural target controllability described in^5,6^. The greedy approach (the algorithm description is in the “Supplementary Information”) to structural constrained target controllability is to build control paths ending in the target nodes and starting in a minimal set of input nodes. The algorithm involves solving an iterated maximum matching problem, based on a graph theoretical result of^41^. The algorithm starts from the target nodes and constructs the control paths by elongating them against the direction of the edges, seeking to minimize the number of new nodes added to the paths in each step. The nodes that can eventually be no longer matched become the input nodes and they are offered as a solution to the structural target controllability problem. The focus of the algorithm is on constructing the control paths connecting the inputs to the targets, and their objective is to minimize the number of input nodes, at the cost of arbitrarily long control paths from the input nodes to the target nodes. In contrast, the genetic algorithm focuses on identifying combinations of nodes in the network that offer a solution to the structural target control problem being solved. It does this in two stages. In the initial stage, a number of solutions are generated by selecting one node from the predecessors of each of the target nodes. To check that such a selection is a solution to the structural target control problem, we use the Kalman rank condition^12^. The search is done within a set distance upstream of the target nodes, to constrain the search space (and implicitly, to constrain the length of the control paths). Any selection of nodes that fails the Kalman rank condition is discarded. In the second stage, the new solutions are generated through combinations of the current solutions and through adding new random solutions to the population. Each new solution is verified for consistency with the Kalman rank condition and discarded if it fails it. Several solutions are also discarded in each step: those with the highest number of input nodes. The size of the population being maintained in the search is a parameter to be set by the modeler; in our tests we used populations of 80 solutions in each iteration of the genetic algorithm. The search strategy of the genetic algorithm is discussed in detail in the “Supplementary Information”.

The greedy algorithm offers a single solution per run, while the genetic algorithm offers several solutions per run: those of minimal size in the population of solutions of the last step of its search. To make the comparison between the greedy and the genetic algorithms balanced, we defined an iteration of the greedy algorithm as a set of 80 independent runs, offering a total of 80 solutions per iteration. Also, to investigate the effect of the pathways being constrained to a certain maximum length (as done in the genetic algorithm), we ran the greedy algorithm in two different settings. In one variant, the maximum path length of the control path was bounded by the same parameter as the genetic algorithm (5, 15, 30, and 50 in different tests, details below). In the other variant, the maximum path length was left unconstrained. Each of the three algorithms (genetic, constrained greedy and unconstrained greedy) was run in this setup 10 times. All the data and the results are available in Supplementary Table 2, and in the application repository at^42^.

### Minimizing the number of input nodes

**Figure 2:**
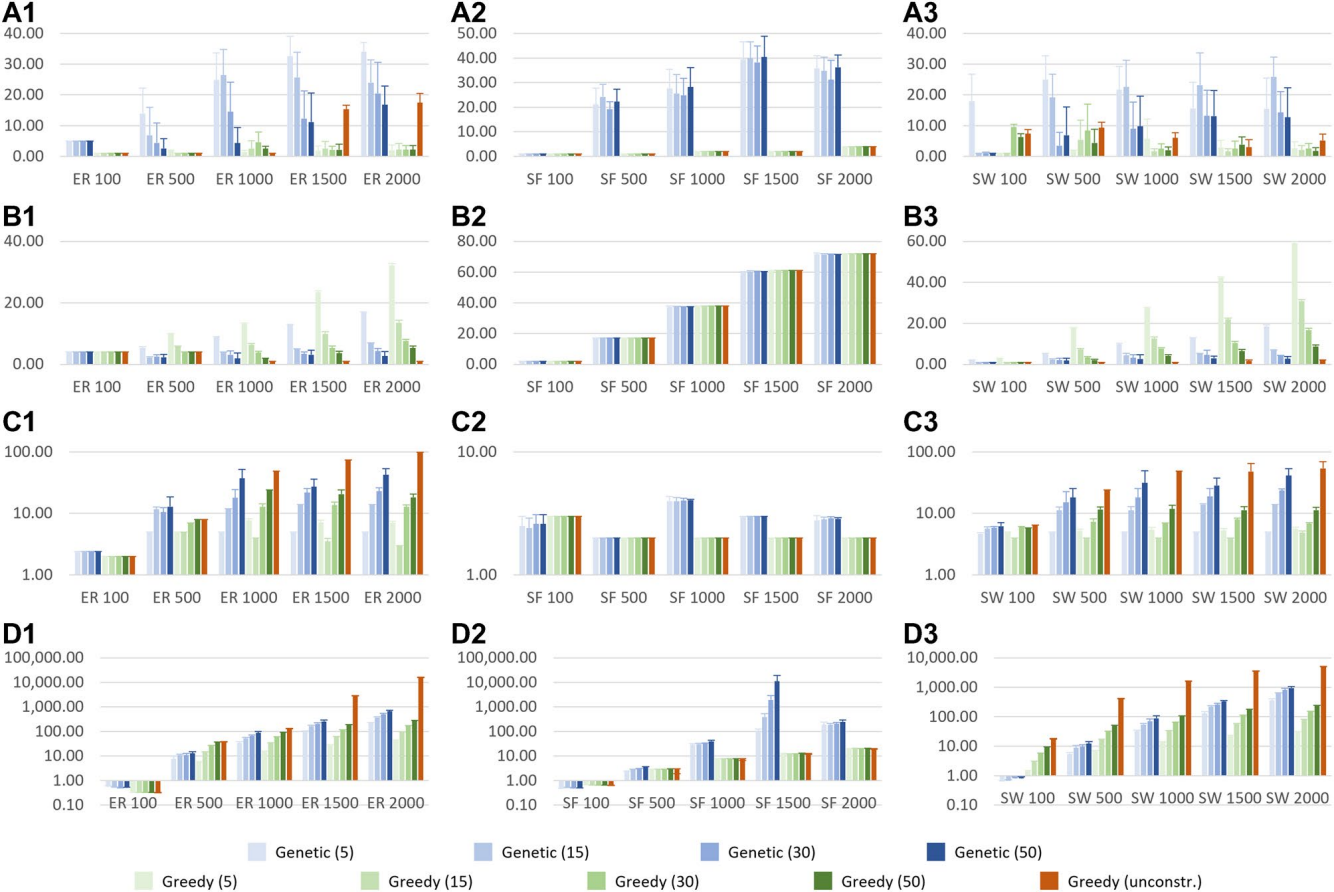
The results of the algorithms on the random networks. For all colors, the darker the shades, the longer are the maximum allowed control paths. (**A**) The number of the identified solutions, (**B**) the size of the solutions, (**C**) the length of the control paths in the solutions, (**D**) running time. Column 1: the Erdős–Rényi networks, column 2: the Scale-Free networks, column 3: the Small World networks. In blue: the results of the genetic algorithm, in green: the results of the constrained greedy algorithm, in orange: the results of the unconstrained greedy algorithm; from left to right, all plots: the networks with 100/500/1000/1500/2000 nodes.

A first benchmark objective that we compared against was the number of distinct solutions identified by each run of the algorithms (repeated runs for greedy, as explained above), and their sizes (number of input nodes). We experimented with different values for the maximum path length (5, 15, 30, and 50), to test the scalability of the methods and the influence of the longer control paths on the size of the solutions. The results are discussed below and presented in detail in Fig. 2A1–C3, and in Supplementary Table 2.

Regarding the number of solutions, (Fig. 2A1–A3) the genetic algorithm identified more solutions than the greedy algorithms in most cases, up to 20 times more in some cases. There were only a few exceptions: in the case of Erdős–Rényi networks with 1500 and with 2000 nodes, the genetic algorithm identified roughly the same number of solutions as the unconstrained greedy, and several times more than constrained greedy. Also, the comparison was inconclusive in the case of the smallest random networks, with only 100 nodes. Obtaining more solutions is a key advantage of the genetic algorithm, especially for large drug repurposing applications, where multiple alternative solutions are important to collect and compare.

Regarding the size of the solutions, (Fig. 2B1–B3) compared to the constrained greedy algorithm, the genetic algorithm identified, on average, solutions 20–50% smaller for the Erdős–Rényi networks, and 50–70% smaller for the Small World networks, for all maximum path length values we experimented with. The differences are minor for the smaller networks, regardless of the allowed maximum path length, but become increasingly notable for the larger networks. The unconstrained greedy algorithm found smaller solutions than the genetic algorithm in the case of the Erdős–Rényi networks and of the Small World networks, at the cost of control paths up to $500\%$ longer, (Fig. 2C1–C3). This is not surprising, because the size of the solutions is closely related to the length of the control paths: the longer the control path, the more targets a single input node can control, so the fewer input nodes are needed to control all targets. When we increased the maximum allowed length of the control paths in the genetic algorithm, the size of its solutions became comparable to that of the unconstrained greedy algorithm. In the case of the Scale-Free networks, the three algorithms identified solutions of roughly the same size. This is because these networks tend to have a small diameter and so, long paths do not exist, eliminating the key advantage of the unconstrained greedy algorithm.

### Running times and convergence speed

Another benchmark objective that we investigated was the total running time and the speed of convergence to a minimal solution. The results are discussed below and presented in detail in Fig. 2D1–D3, and in Supplementary Table 2.

The genetic algorithm had a comparable running time to the constrained greedy algorithm, from 2 times slower to 2 times faster, and was up to 500 times faster than the unconstrained greedy algorithm for the Erdős–Rényi and Small World networks. On the other hand, in the case of the sparser-connected Scale-Free network, the greedy algorithms were considerably faster than the genetic one. The reason for the greedy algorithm being faster on these networks is that fewer edges implies fewer options to test when building the control paths, hence a faster running time. The Scale-Free networks tend to have a small number of “hubs” (highly connected nodes) and this leads to a difficulty with the genetic algorithm: such hubs may be considered as potential control inputs for many targets (because of their high number of descendants in the networks), but the Kalman condition will fail anytime they are suggested as control inputs for two or more targets at the same distance from the hub.

We analyzed the evolution of the quality of the solutions throughout the ten iterations of each of the algorithms, and we noticed that the optimal solutions were found very quickly, typically within the first three iterations. Moreover, within one iteration of the genetic algorithm, a near-optimal solution (i.e., a solution with the size within $10\%$ of the best solution) was achieved very quickly, typically within 80 generations from the start for the Erdős–Rényi and Small World networks, and within only ten generations for the Scale-Free networks (Fig. S2). This suggests that the genetic algorithm may be applied successfully with a much lower number of generations, adding a considerable speed-up to it; by default, in our study we used a default number of 100 consecutive generations without an improvement in the size of the optimal solutions.

### Maximizing the use of preferred inputs

**Figure 3:**
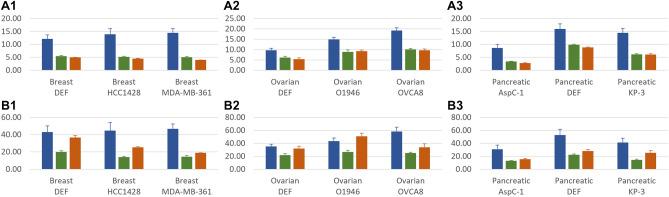
The results of the algorithms on the biological networks. (**A**) Number of identified drug-targetable inputs, (**B**) number of targets controllable from the identified drug-targetable inputs; column 1: breast cancer networks, column 2: ovarian cancer networks, column 3: pancreatic cancer networks; in blue: (constrained) genetic algorithm, in green: constrained greedy algorithm, in orange: unconstrained greedy algorithm.

We compared the ability of the three algorithms to maximize the use of preferred nodes, and we performed a brief literature-based validation of the relevance of the drug-targets and drugs found for the cancer networks. The results are discussed below and presented in detail in Fig. 3, and in Supplementary Table 2.

We applied the three algorithms on our benchmark biological networks with the additional optimization objective of maximizing the selection of FDA-approved drug targets as preferred input nodes. In the greedy algorithm, maximizing the use of FDA-approved drug targets is implemented in every step of extending the control paths. All nodes acting as starting points in the control paths being constructed are attempted to be matched with preferred nodes; the nodes left unmatched are then attempted to be matched with un-preferred nodes; finally, the nodes that could not be matched towards longer control paths are selected as input nodes and their control path is completed. In the genetic algorithm, maximizing the use of preferred nodes is done when selecting the potential input nodes in every candidate solution. The choice is stochastic with a larger probability to be selected set for the preferred nodes. In our experiments we used a probability of 2/3 for the preferred nodes to be selected in a candidate solution and a probability of 1/3 for the un-preferred nodes. The optimal balance between the two probabilities is a function of the balance between preferred and un-preferred nodes in the network, and of their out-degrees. Too high a probability for preferred nodes may make it difficult to get a full rank controllability matrix (i.e., to control the entire target set) and many attempts may have to be made before one is found; too low a probability for them will lead to a sub-optimal solution in terms of how many preferred nodes are eventually included in the optimal solution.

For all networks and for all experiments we ran, the sets of input nodes returned by the genetic algorithm contained, on average, 150–300% more preferred nodes than the ones returned by either of the greedy algorithms. This led to 120–320% more control target nodes being controlled by preferred nodes in all but one cases, i.e., leading to predictions of potentially more efficient drugs. This has as a consequence a clear improvement in the applicability of the algorithm in the biomedical domain for drug repurposing, an aspect that we discuss in the next subsection.

**Figure 4:**
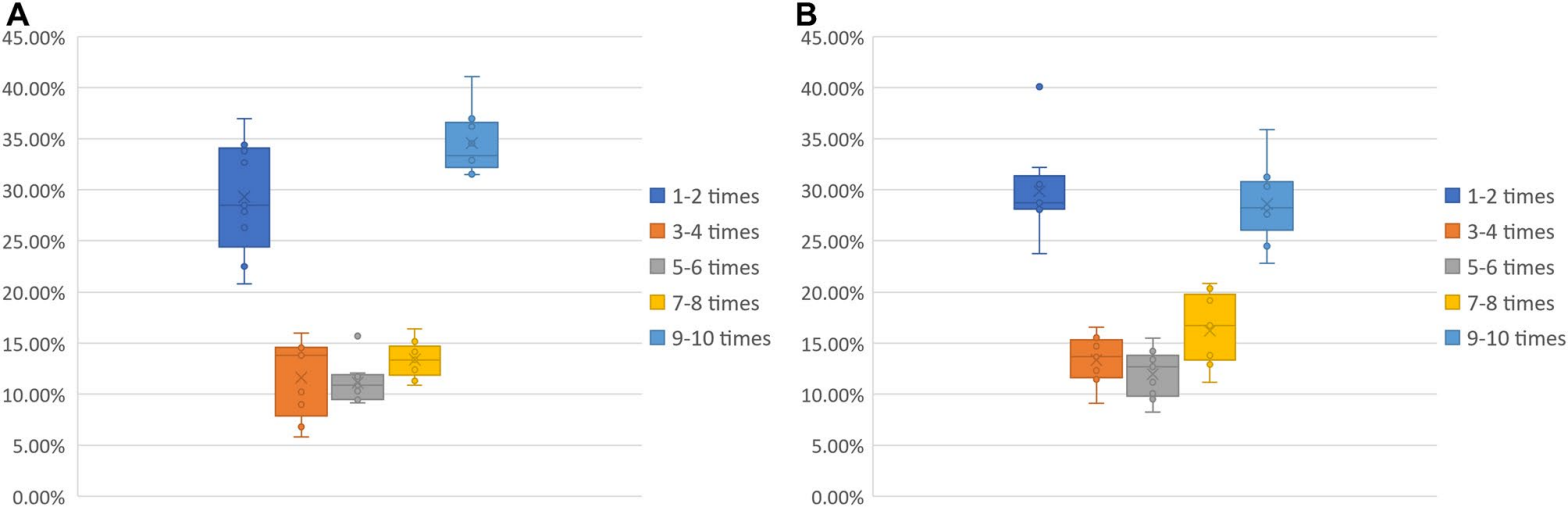
The distribution of input nodes repeatedly identified over 10 iterations of the genetic algorithm for the biological networks. For each biological network we counted how many of the input nodes were found in at least 9–10/7–8/5–6/3–4/1–2 of the 10 runs we did on that network. The box plots show the distribution of these counts over all the biological networks. (**A**) Repeated (stochastic) runs over the biological networks. (**B**) Repeated runs over the biological networks with of their $5\%$ edges randomly removed.

To test the reproducibility of the results and the robustness of the genetic algorithm, we investigated how often the preferred nodes are identified over multiple runs of the algorithm. We performed these tests within two setups. First, we ran the algorithm 10 times on the benchmark biological networks; with the algorithm being stochastic, there was a diversity of solutions offered from run to run. Second, we ran the algorithm 10 times more, each time on slightly modified networks, with a random set of $5\%$ edges removed for each iteration, to emulate the effect of false positives in the interaction data. The results are presented in Fig. 4. In both cases, about $50\%$ of the preferred nodes were consistently identified in at least 7 of the 10 runs, and about $30\%$ were identified in at least 9 of the 10 runs.

### Therapeutically relevant results

**Figure 5:**
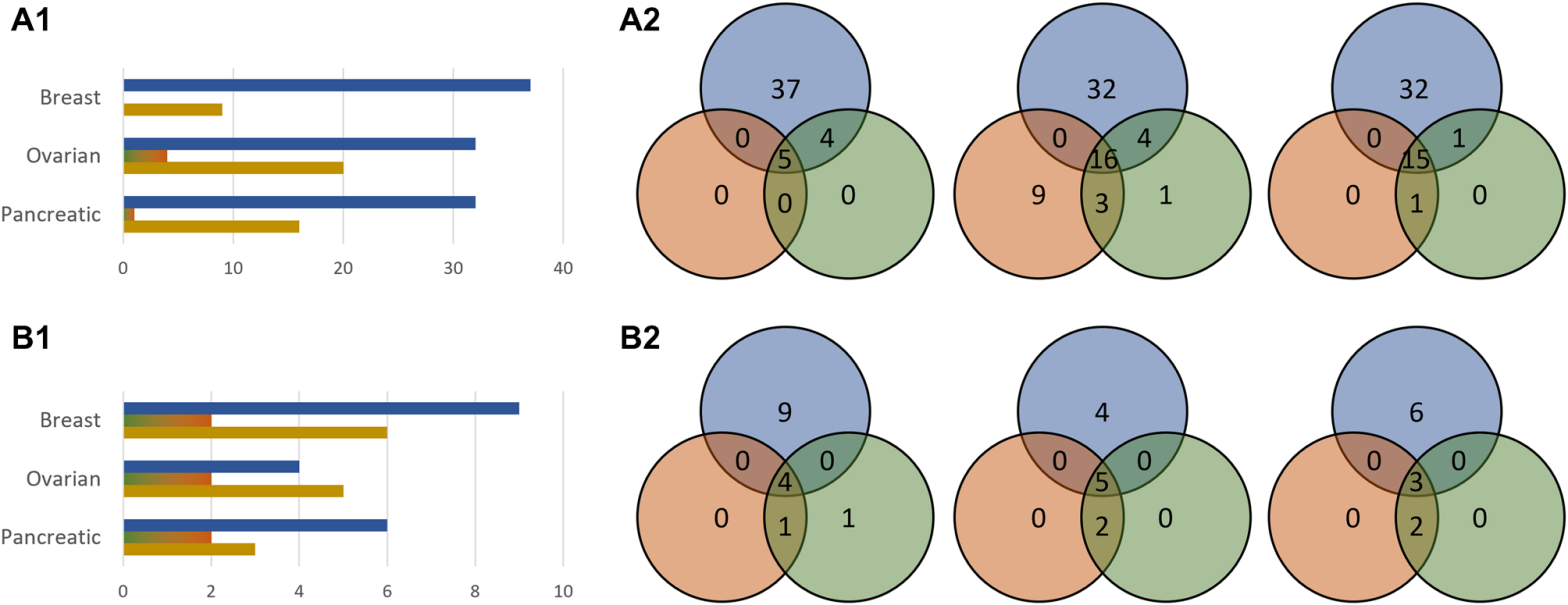
The identified drug-targets over repeated iterations for each algorithm. (**A**) Considering the drug-targets identified in at least one iteration by at least one algorithm, (**B**) considering the drug-targets identified in at least half of the iterations by at least one algorithm; column 1: specific-algorithm identified drug-targets, column 2: overlap between identified drug-targets by algorithm; in blue: (constrained) genetic algorithm, in green: constrained greedy algorithm, in orange: unconstrained greedy algorithm, in yellow: the intersection of all algorithms.

We performed a brief analysis of the top FDA-approved drug targets identified in multiple runs of the algorithms for each of the biological networks. We used the DrugBank database^38^ to find approved and investigational drugs targeting these proteins and known to be used in cancer therapeutics. To avoid spurious selections of inputs, for each cancer type, we considered only the drug targets that have been identified in at least half of the runs of one of the three algorithms. The sets of drug-targets returned by the unconstrained and constrained versions of the greedy algorithms and their corresponding drugs have been combined for a better comparison against the genetic algorithm. Even so, for all the cancer networks we analyzed, the genetic algorithm managed to identify more than twice drug-targets and cancer-related drugs than its counterparts. With only one exception, all of these drug-targets are known to be of significance in the corresponding cancer type. The results are presented in Table 2, in Fig. 5, and in Supplementary Table 3.

**Table 2:** The intersection and the algorithm-specific predicted drug-targets and corresponding drugs.

Type	Algorithm	Drug-targets	Drugs
Breast	Genetic	VDAC1$^{\star}$^43^ ($100.00\%$), DDR1$^{\star }$^44^ ($100.00\%$), ALK$^{\star }$^45^ ($100.00\%$), SRC$^{\star }$^46^ ($90.00\%$), JAK2$^{\star }$^47^ ($86.67\%$), FGFR4$^{\star }$^48^ ($66.67\%$), IL3RA$^{\dagger }$^49^ ($63.33\%$), LCK$^{\star }$^50^ ($56.67\%$), FGF4$^{\star }$^51^ ($53.33\%$)	alectinib$^{{\dagger },{\ddagger }}$^52,53^, bosutinib$^{{\dagger },{\ddagger }}$^54,55^, ceritinib$^{\dagger }$^56^, crizotinib${^{{\star },{\ddagger }}}$^57,58^, dasatinib${^{{\star },{\ddagger }}}$^59,60^, entrectinib$^{{\dagger },{\ddagger }}$^52,61^, gliteritinib$^{\dagger }$^62^, infigratinib$^{{\dagger },{\ddagger }}$^63,64^, lenvatinib${^{{\star },{\ddagger }}}$^65,66^, lorlatinib$^{\dagger }$^67^, nintedanib${^{{\star },{\ddagger }}}$^68,69^, pemigatinib$^{{\dagger },{\ddagger }}$^52,70^, ponatinib$^{{\dagger },{\ddagger }}$^52,71^, pralsetinib$^{\dagger }$^72^
	Greedy	IGF1R$^{\star }$^73^ ($100.00\%$), AKT1$^{\star }$^74^ ($30.00\%$)	cixutumumab$^{{\dagger },{\ddagger }}$^75,76^, genistein${^{{\star },{\ddagger }}}$^77,78^, arsenic trioxide$^{\star }$^79^
	Both	KEAP1$^{\star }$^80^ ($100.00\%$), PIM1$^{\star }$^81^ ($98.89\%$), IL3$^{\dagger }$^82^ ($83.33\%$), DDR2$^{\star }$^83^ ($62.22\%$), RET$^{\star }$^84^ ($46.67\%$), EGFR$^{\star }$^85^ ($44.44\%$)	afatinib${^{{\star },{\ddagger }}}$^86,87^, alvocidib${^{{\star },{\ddagger }}}$^88,89^, amivantamab$^{\dagger }$^90^, *AV-412*^91^, brigatinib$^{{\dagger },{\ddagger }}$^52,92^, cetuximab$^{{\dagger },{\ddagger }}$^93,94^, dacomitinib$^{\star }$^95^, erdafitinib$^{{\dagger },{\ddagger }}$^96,97^, erlotinib${^{{\star },{\ddagger }}}$^52,98^, gefitinib$^{{\dagger },{\ddagger }}$^99,100^, icotinib$^{\dagger }$^101^, imatinib$^{{\dagger },{\ddagger }}$^102,103^, lapatinib${^{{\star },{\ddagger }}}$^52,104^, matuzumab$^{\dagger }$^105^, mobocertinib$^{\dagger }$^106^, necitumumab$^{\dagger }$^107^, neratinib${^{{\star },{\ddagger }}}$^108,109^, olmutinib$^{\dagger }$^110^, osimertinib$^{\dagger }$^111^, panitumumab$^{{\dagger },{\ddagger }}$^112,113^, pelitinib$^{\dagger }$^114^, regorafenib$^{\star }$^115^, vandetanib${^{{\star },{\ddagger }}}$^116,117^, varlitinib${^{{\star },{\ddagger }}}$^118,119^, XL228$^{\dagger }$^120^, zanubrutinib$^{\dagger }$^121^
Ovarian	Genetic	DDR1$^{\star }$^122^ ($100.00\%$), PDPK1$^{\dagger }$^123^ ($63.33\%$), SRC$^{\star }$^124^ ($56.67\%$), ERBB2$^{\star }$^125^ ($53.33\%$)	afatinib$^{\star }$^126^, *AV-412*^91^, bosutinib$^{{\dagger },{\ddagger }}$^55,127^, brigatinib$^{\dagger }$^92^, dasatinib${^{{\star },{\ddagger }}}$^128,129^, imatinib${^{{\star },{\ddagger }}}$^130,131^, lapatinib$^{{\dagger },{\ddagger }}$^104,132^, margetuximab$^{\dagger }$^133^, nintedanib${^{{\star },{\ddagger }}}$^134,135^, pertuzumab${^{{\star },{\ddagger }}}$^136,137^, ponatinib$^{\star }$^138^, pralsetinib$^{\dagger }$^72^, trastuzumab$^{\star }$^139^, trastuzumab emtansine$^{{\dagger },{\ddagger }}$^140,141^, tucatinib$^{\dagger }$^142^, XL228$^{\dagger }$^120^, zanubrutinib$^{\dagger }$^121^
	Greedy	HCK$^{\dagger }$^143^ ($66.67\%$), FGF1$^{\star }$^144^ ($63.33\%$)	abemaciclib${^{{\star },{\ddagger }}}$^145,146^, alvocidib${^{{\star },{\ddagger }}}$^147,148^, palbociclib${^{{\star },{\ddagger }}}$^149,150^, ribociclib$^{{\dagger },{\ddagger }}$^151,152^
	Both	PIM1$^{\star }$^153^ ($95.56\%$), SMO$^{\star }$^154^ ($92.22\%$), DDR2$^{\star }$^155^ ($78.89\%$), PRKDC$^{\dagger }$^156^ ($66.67\%$), CCL2$^{\star }$^157^ ($65.56\%$)	glasdegib$^{\dagger }$^158^, regorafenib$^{{\dagger },{\ddagger }}$^159,160^, SF1126$^{\dagger }$^161^, sonidegib$^{{\dagger },{\ddagger }}$^158,162^, vismodegib$^{{\dagger },{\ddagger }}$^158,163^
Pancreatic	Genetic	IGF1R$^{\star }$^164^ ($73.33\%$), ADCY1 ($66.67\%$), SRC$^{\star }$^165^ ($60.00\%$), PDPK1$^{\star }$^166^ ($53.33\%$), AKT1$^{\star }$^167^ ($53.33\%$), MTOR$^{\star }$^168^ ($50.00\%$)	arsenic trioxide${^{{\star },{\ddagger }}}$^169,170^, cixutumumab$^{\star }$^171^, everolimus${^{{\star },{\ddagger }}}$^172,173^, genistein$^{\star }$^174^, nintedanib${^{{\star },{\ddagger }}}$^175,176^, ridaforolimus$^{\dagger }$^177^, SF1126$^{\dagger }$^161^, temsirolimus${^{{\star },{\ddagger }}}$^178,179^, XL765$^{\dagger }$^180^
	Greedy	GSK3B$^{\star }$^181^ ($66.67\%$), CDK4$^{\star }$^182^ ($66.67\%$)	pazopanib$^{\dagger }$^183^
	Both	CDK2$^{\star }$^184^ ($85.56\%$), ABL1$^{\dagger }$^185^ ($73.33\%$), PIM1$^{\star }$^186^ ($66.67\%$)	alvocidib$^{{\dagger },{\ddagger }}$^147,187^, AT-7519$^{\star }$^188^, bosutinib$^{\dagger }$^189^, brigatinib$^{\dagger }$^92^, dasatinib${^{{\star },{\ddagger }}}$^190,191^, imatinib$^{{\dagger },{\ddagger }}$^192,193^, nilotinib$^{\dagger }$^194^, ponatinib$^{\dagger }$^71^, radotinib$^{\dagger }$^195^, regorafenib$^{{\dagger },{\ddagger }}$^159,196^, seliciclib$^{\dagger }$^197^, umbralisib$^{\dagger }$^198^, XL228$^{\dagger }$^120^

Only the drug-targets that have been identified in at least half of the iterations for at least one algorithm have been considered. In brackets: percentage of individual iterations in which the drug-target was identified for the corresponding cancer type; with a$^{\star }$: proteins known to be of significance in the corresponding cancer type and drugs investigated for or used in the treatment of the corresponding cancer type, with a$^{\dagger }$: proteins known to be of significance in other or unspecified cancer types and drugs investigated for or used in the treatment of other or unspecified cancer types, with a$^{\ddagger }$: drugs investigated for the treatment of the corresponding cancer type within active or completed clinical trials.

Out of the nine top preferred inputs identified solely by the genetic algorithm for the breast cancer networks, eight are known to be of significance in breast cancer proliferation: VDAC1^43^, DDR1^44^, ALK^45^, SRC^46^, JAK2^47^, FGFR4^48^, LCK^50^, and FGF4^51^. In addition, there are six more preferred inputs identified by both the genetic and by the greedy algorithms, out of which five are known as important in breast cancer: KEAP1^80^, PIM1^81^, DDR2^83^, RET^84^, and EGFR^85^. Furthermore, the remaining two top preferred inputs are known to be of significance in other cancer types: IL3RA^49^ and IL3^82^, marking them as potential drug repurposing targets for future research. The greedy algorithms returned only two specific preferred inputs not found by the genetic algorithm, both known to be significant for breast cancer: IGF1R^73^ and AKT1^74^. We found fourteen cancer-related drugs which are targeting inputs only identified by the genetic algorithm, four of which have been investigated for the treatment of breast cancer (crizotinib^57^, dasatinib^59^, lenvatinib^65^, and nintedanib^68^), and ten drugs used in an active or in a completed clinical trial (alectinib^52^, bosutinib^54^, crizotinib^58^, dasatinib^60^, entrectinib^52^, infigratinib^63^, lenvatinib^66^, nintedanib^69^, pemigatinib^52^, and ponatinib^52^). In addition, 26 drugs are targeting inputs identified by both algorithms, out of which the effect of nine has been studied for the treatment of breast cancer, and thirteen used in a clinical trial. In comparison, we found only three cancer-related existing drugs targeting the inputs only identified by the greedy algorithms, all of which are investigated in the treatment of, or under clinical investigation, for breast cancer.

The results look similar for the analyzed ovarian cancer networks. The genetic algorithm identified four specific top preferred inputs, out of which three are known to be of significance in ovarian cancer: DDR1^122^, SRC^124^, and ERBB2^125^. In contrast, only two were found by the greedy algorithms and not by the genetic algorithm, with one of significance in ovarian cancer: FGF1^144^. Both remaining inputs, one for each algorithm, are known to be important in other cancer types: PDPK1^123^ and HCK^143^. There are five additional drug-targets that have been identified by both algorithms, four of which of importance in ovarian cancer: PIM1^153^, SMO^154^, DDR2^155^, and CCL2^157^, and one in other types of cancer: PRKDC^156^. Of the related drug-targets, we found seventeen that are targeting the inputs identified only by the genetic algorithm, with seven under research for the treatment of ovarian cancer (afatinib^126^, dasatinib^128^, imatinib^130^, nintedanib^134^, pertuzumab^136^, ponatinib^138^, trastuzumab^139^), and seven part of an active or completed clinical trial (bosutinib^127^, dasatinib^129^, imatinib^131^, lapatinib^132^, nintedanib^135^, pertuzumab^137^, and trastuzumab emtansine^140^). Five more drugs are targeting inputs identified by both algorithms, with three of them part of a clinical trial. We found only four cancer-related drugs specific to the greedy algorithms, all of which are either researched, or under clinical investigation for ovarian cancer.

The results are consistently in favor of the genetic algorithm also in the case of the pancreatic cancer networks. There are three preferred inputs identified by both algorithms, two of which of importance in pancreatic cancer: CDK2^184^, and PIM1^186^, and one in other types of cancer: ABL1^185^. The genetic algorithm found six specific drug-targets, with five significant in pancreatic cancer: IGF1R^164^, SRC^165^, PDPK1^166^, AKT1^167^, and MTOR^168^, as opposed to only two for the greedy algorithms, both of significance in pancreatic cancer: GSK3B^181^, CDK4^182^. We found ten drugs specific to the genetic algorithm, out of which six investigated for the treatment of pancreatic cancer (arsenic trioxide^169^, cixutumumab^171^, everolimus^172^, genistein^174^, nintedanib^175^, and temsirolimus^178^), and four under clinical trials (arsenic trioxide^170^, everolimus^173^, nintedanib^176^, and temsirolimus^179^). In contrast, we found only one drug specific to the greedy algorithm. We found thirteen further drugs common to both algorithms, with two being researched, and four under clinical investigation for pancreatic cancer. The genetic algorithm uniquely identified ADCY1, currently not known to be targeted by any cancer-related drugs.

Furthermore, we found the drug fostamatinib, used for the treatment of rheumatoid arthritis and immune thrombocytopenic purpura^199^, which targets multiple inputs identified by the genetic algorithm in multiple runs for all studied cancer networks. Our algorithm thus suggests that the drug could potentially be used in cancer treatment. This idea is supported by several completed clinical trials for using fostamatinib in treating lymphoma^200^ and is investigated in an ongoing trial for ovarian cancer^201^.

## Discussion

Applying network controllability for drug repurposing is not yet well-established. It is a very promising line of research that is currently hindered in part by the lack of powerful implementations of network controllability algorithms. This is where this paper and its algorithm contribute, making it possible to run detailed drug repurposing studies based on network controllability. We applied the algorithm to a few relatively small cancer examples, demonstrating its feasibility in the medical domain. Our demonstration was intended to show the potential of the network controllability approach to help in such studies. Our case studies suggest that this approach is promising in drug repurposing: we identified several approved drugs whose targets contribute to controlling the essential genes specific to other diseases than those the drugs were approved for. These results are to be considered as proof-of-concept, rather than fully fledged validated demonstrations of network control-based drug repurposing identification. Our search strategy was based on a genetic algorithm, where the population in each generation of the training of the algorithm is a set of valid solutions to the network controllability problem. The algorithm turned out to be scalable, with its performance staying strong even for large networks.

Our approach is to integrate the global interaction data into the directed networks and investigate a global optimization problem seeking to minimize the number of input nodes needed to control a target set. Genetic algorithms represent a known global optimization technique that permits for a global search of solutions throughout all parts of the network. They have been successfully used for solving combinatorial optimization problems, performing potentially better when compared to greedy algorithms^23^. Their use comes with limitations that we addressed in our implementation. To start with, evaluating the fitness function can generally be very computationally expensive. Within our approach, in order to calculate the fitness of a chromosome we first need to check its validity, which requires establishing if its corresponding Kalman matrix, whose size is dependent on the size of the target set, has a full rank. This operation can be computationally expensive for large matrices. We included in the “Supplementary Information” an overview of the function we used and its complexity. Another limitation refers to the genetic algorithms’ proneness to converging to a local optimum or arbitrary points, as opposed to the global optimum. To address this, we insert in each stage new random chromosomes (in addition to adding chromosomes obtained through crossover and mutation), to enrich the search space of the algorithm and escape the local optima. We also use elitism to ensure that each generation contains the distinct best solutions identified so far by the algorithm. Moreover, another limitation of the genetic algorithm is represented by the fact that the optimal result is not known and the quality of a solution is only comparable to the other solutions within a run. Thus, we alleviate the lack of a definite stopping criteria by running iteratively, each time until no new solutions have been identified within a predefined number of generations; by default, we set this to 100 generations.

The genetic algorithm comes by design with a set-limit on the maximum length of control paths from the input to the target nodes. We made this into a parameter whose value can be set by the user. Having this parameter is a feature of particularly important interest in applications in medicine, where the effects of a drug dissipate quickly over longer signaling paths. The focused search upstream of the target nodes led to the genetic algorithm drastically improving the percentage of FDA-approved drug targets selected in its solution, a clear step forward towards applications in combinatorial drug selection and drug repurposing. The drugs identified by our algorithm as potentially efficient for breast, ovarian, and pancreatic cancer correlate well with recent literature results, and some of our suggestions have already been subject to several clinical studies. This strengthens the potential of our approach for studies in synthetic lethality-driven drug repurposing.

There are several interesting questions around the applications of network science to drug repurposing, that deserve further investigations. On the experimental side, validating the predictions made by the network-based approaches would help drive this research line forward, and it would offer an insight into how to apply it to disease data. Some demonstrations already exist^19^, but more work is needed before the network analytics approach becomes a standard tool in this field. On the computational side, adding to the framework some quantitative aspects about the type and the weight of the interactions would help eliminate some of the false positive results. Also, adding non-linear interactions to the models would help extend the applicability of this method. A lot of work in this direction has already been done, e.g. on Boolean networks^202–204^, but the methods still suffer from scalability issues. Very large networks, with tens of thousands of interactions, are already practical based on linear network analyses, including the genetic algorithm proposed in this study.

## Methods

We introduce briefly in the “Supplementary Information” the basic concepts of target structural network controllability and the Kalman matrix rank condition for the problem.

The algorithm takes as input a network given as a directed graph $G=(V,E)$ and a list of target nodes $T\subseteq V$, $T=\{t_1,\ldots ,t_l\}$. We denote the graph’s adjacency matrix by $A_G$. The algorithm gives as a result a set of input nodes $I\subseteq V$ controlling the set *T*, with the objective being to minimize the size of *I*. The algorithm can also take as an additional, optional input a set $P\subseteq V$ of so-called preferred nodes. In this case, the algorithm will aim for a double optimization objective: minimize the set *I*, while maximizing the number of elements from *P* included in *I*. Our typical application scenario is that of a network *G* consisting of directed protein-protein interactions specific to a disease mechanism of interest, with the set of targets *T* being a disease-specific set of essential genes, and the set of preferred nodes *P* a set of proteins targetable by available drugs or by specially designed compounds (e.g., inhibitors, small silencing molecules, etc.) The terminology we use to describe the algorithm, e.g., population/chromosome/crossover/mutation/fitness is standard in the genetic algorithm literature and refers to its conventions, rather than being suggestive of specifics in molecular biology.

Our algorithm starts by generating several solutions to the control problem, in the form of several “control sets” $I_1, \ldots , I_m$; we discuss how this is achieved in the “Supplementary Information”. Each such solution is encoded as a “chromosome”, i.e., a vector of “genes” $[g_1,\ldots ,g_l]$, where for all $1\le i\le l$, $g_i\in V$ controls the target node $t_i\in T$. In particular, $g_i$ is an ancestor of $t_i$ in graph *G*, for all $1\le i\le l$. Note that a node can control simultaneously several other nodes in the network and so, the genes $g_1,\ldots ,g_l$ of a chromosome are not necessarily distinct. In fact, the fewer the genes on a chromosome, the better its fitness will be, as we discuss in the “Supplementary Information”. The algorithm also implements maximizing the use of preferred nodes as genes on the chromosomes; the details are discussed in the “Supplementary Information”.

A set of chromosomes is called a population. Note that a chromosome will always encode a solution to our optimization problem, throughout the iterative run of the algorithm. Any population maintained by the algorithm consists of several such chromosomes, some better than others from the point of view of our optimization criteria, but all valid solutions to the target controllability problem to be solved.

The algorithm iteratively generates successive populations (sets of chromosomes) that get better at the optimization it aims to solve: the number of distinct genes on some chromosomes gets smaller and the proportion of preferred nodes among them gets higher. The algorithm stops after a number of iterations in which the quality of the solutions does not improve. To have a bounded running time even for large networks, we also added a stop condition after a maximum number of iterations; this was never reached during our tests. This pre-defined stop is necessary since the target structural controllability problem is known to be NP-hard and so, finding the optimal solution can require a prohibitively high number of steps, potentially exponential in the number of nodes in the network. The output consists of several solutions to the problem, represented by all the control sets in the final population obtained by the algorithm.

The initial population of solutions is randomly generated in such a way that each element selected for it is a solution to the target structural controllability problem $(A_G,I,T)$. To generate the next generation from the current one, we use three techniques:

- Retain in the population the best solutions (from the point of view of the assessed optimization problem, as encoded in the fitness function). “Elitism” is used to conserve the best solutions (further discussed in the “Supplementary Information”).
- Add random chromosomes (all being valid solutions to the optimization problem, albeit potentially of lower fitness score than some of the others in the population).
- Generate new solutions/chromosomes resulting from combinations of those in the current population. A selection operator is used to choose the chromosomes which will produce offsprings for the following generation. New chromosomes are produced using crossover and mutation (discussed in detail in the “Supplementary Information”).

A list of all the parameters used by the genetic algorithm can be found in Table 3. The basic outline of the proposed genetic algorithm is described below. All operators are detailed in the “Supplementary Information”.

1. Generate the initial population: we set $t \leftarrow 0$ for the first generation. We initialize *P*(*t*) with a number of *n* randomly generated chromosomes.
2. Preserve the fittest chromosomes: we evaluate the fitness of all chromosomes in *P*(*t*). We add to the next population $P_{t+1}$ the $p_e \cdot n$ chromosomes in the current generation with the highest fitness score, where $0\le p_e<1$ is the ‘elitism’ parameter. If there are more chromosomes of equal fitness, the ones to be added are randomly chosen.
3. Add random chromosomes: we add $p_r \cdot n$ new randomly generated chromosomes to $P(t + 1)$, where $0\le p_r<1$ is the ‘randomness’ parameter.
4. Add the offsprings of the current population: we apply two times the selection operator on *P*(*t*), obtaining two chromosomes of $P_t$ selected randomly with a probability proportional to their fitness score. On the two selected chromosomes we apply the crossover operator, obtaining an offspring to be added to $P(t + 1)$. The offspring is added in a mutated form with the mutation probability $0\le p_m<1$. We continue applying this step until the number of chromosomes in $P(t + 1)$ becomes *n*.
5. Iterate: if the current index $t<N$, the maximum number of generations, we set $t \leftarrow t + 1$ and continue with Step 2.
6. Output: we return the fittest chromosomes in the current generation as solutions to the problem and stop.

**Table 3:** The parameters used by the genetic algorithm.

Parameter	Meaning	Default
$N \in \mathbb {N}_{\ge 1}$	Total number of generations for which the algorithm runs	*N* = 10,000
$N' \in \mathbb {N}_{\ge 1}$	Number of generations for which the algorithm runs without improving the fitness of the best chromosomes	$N' = 100$
$n \in \mathbb {N}_{\ge 2}$	Total number of chromosomes in a generation	$n = 80$
$p_m \in \mathbb {R}_{[0, 1]}$	Probability of mutation for a chromosome	$p_m = 0.01$
$p_e \in \mathbb {R}_{[0, 1]}$	Maximum percentage of elites in a generation	$p_e = 0.25$
$p_r \in \mathbb {R}_{[0, 1]}$	Percentage of randomly generated chromosomes in a generation	$p_r = 0.25$
${\text {max}}_{path} \in \mathbb {N}_{\ge 1}$	Max. number of interactions in a control path	${\text {max}}_{path} = 5$
${\text {max}}_{rand} \in \mathbb {N}_{\ge 1}$	Max. number of randomly generated genes in a chromosome	${\text {max}}_{rand} = 15$

## Supplementary Information

Supplementary Information.

Supplementary Table 1.

Supplementary Table 2.

Supplementary Table 3.

## Data Availability

All of the data and algorithm results are available in the application repository at^42^.
