# Supplementary material for: Network controllability solutions for computational drug repurposing using genetic algorithms
Source: Sci Rep. 2022 Jan 26;12:1437. doi: 10.1038/s41598-022-05335-3 (PMC8791995; doi:10.1038/s41598-022-05335-3)
Supplement: Supplementary file 1 — Supplementary Information. [file 41598_2022_5335_MOESM1_ESM.pdf]

Supplementary Information: Network controllability solutions for computational drug repurposing using genetic algorithms

Victor-Bogdan Popescu[1], Krishna Kanhaiya[1], Dumitru Iulian Năstac[2], Eugen Czeizler[1,4], and Ion Petre[3,4,*]

[1] Åbo Akademi University, Computer Science, Turku, 20500, Finland
[2]POLITEHNICA University of Bucharest, Faculty of Electronics, Telecommunications and Information Technology, Bucharest, 061071, Romania
[3]University of Turku, Department of Mathematics and Statistics, Turku, 20014, Finland
[4]National Institute for Research and Development in Biological Sciences, Bucharest, 060031, Romania
[*]Correspondence to: ion.petre@utu.fi

ABSTRACT

Control theory has seen recently impactful applications in network science, especially in connections with applications in network medicine. A key topic of research is that of finding minimal external interventions that offer control over the dynamics of a given network, a problem known as network controllability. We propose in this article a new solution for this problem based on genetic algorithms. We tailor our solution for applications in computational drug repurposing, seeking to maximize its use of FDA-approved drug targets in a given disease-specific protein-protein interaction network. We show how our algorithm identifies a number of potentially efficient drugs for breast, ovarian, and pancreatic cancer. We demonstrate our algorithm on several cancer networks and on several random networks with their edges distributed according to the Erdős-Rényi, the scale-free, and the small world properties. Overall, we show that our new algorithm is more efficient in identifying relevant drug targets in a disease network, advancing the computational solutions needed for new therapeutic and drug repurposing approaches.

Network controllability

We introduce briefly the basic concepts of network controllability and the Kalman condition for the target controllability problem. For more details we refer to[1]. By convention, all vectors are considered to be column vectors so that the matrix-vector multiplications are well defined.

Let $A \in \mathbb{R}^{n\times n}$ be an $n \times n$ matrix, for some $n > 1$. The linear dynamical system defined by matrix A is an n-dimensional vector x of real functions, $x : \mathbb{R} \to \mathbb{R}^n$, defined as the solution of the system of ordinary differential equations

$$\frac{dx(t)}{dt} = Ax(t), \tag{1}$$

for some given $x(0)$. The structure of a linear dynamical system can be thought of as an edge-labeled directed graph with n vertices and adjacency matrix A: for any nodes i, j, $1 \le i, j \le n$, the (directed) edge (i, j) documents node i being influenced by node j, with the weight/label of the influence given by the (i, j) entry $a_{i,j}$ of matrix A.

We consider a subset of input nodes $I \subseteq \{1,2,\ldots,n\}$, $I = \{i_1,\ldots,i_m\}$, $m \le n$, thought of as the nodes of the linear dynamical system on which an external contribution can be applied to influence the dynamics of the system. The subset I can also be described through its characteristic matrix $B_I \in \mathbb{R}^{n\times m}$, defined as $B_I(r,s) = 1$ if $r = i_s$ and 0 otherwise, for all $1 \le r \le n$ and $1 \le s \le m$. The external influence is exerted through an m-dimensional input vector u of real functions, $u : \mathbb{R} \to \mathbb{R}^m$. The influence of the input vector u on the linear dynamical system is described by the equation

$$\frac{dx(t)}{dt} = Ax(t) + B_I u(t). \tag{2}$$

We also consider a subset of target nodes $T \subseteq \{1,2,\ldots,n\}$, $T = \{t_1,\ldots,t_l\}$, $m \le l \le n$, thought of as a subset of the nodes of the linear dynamical system whose dynamics we aim to control (as defined below) through a suitable choice of input nodes

and of an input vector. The subset of target nodes can also be defined through its characteristic matrix $C_T \in \mathbb{R}^{l\times n}$, defined as follows: $C_T(r,s) = 1$ if $t_r = s$ and $C_T(r,s) = 0$ otherwise, for all $1 \leq r \leq l$ and $1 \leq s \leq n$.

The triplet (A,I,T) is called the targeted linear dynamical system with inputs, defined by matrix A, input set I and target set T. We say that this system is target controllable if for any $x(0) \in \mathbb{R}^n$ and any $\alpha \in \mathbb{R}^l$, there is an input vector $u : \mathbb{R} \to \mathbb{R}^m$ such that the solution $\tilde{x}$ of (2) eventually coincides with α on its T-components, i.e., $C_T\tilde{x}(\tau) = \alpha$, for some $\tau \geq 0$. Intuitively, the system being target controllable means that for any input state x_0 and any desired final state α of the target nodes, there is a suitable input vector u driving the target nodes to α. Obviously, the input vector u depends on x_0 and α.

The question whether a given targeted linear dynamical system with inputs (A,I,T) is controllable has an elegant algebraic answer known as the Kalman condition.

Theorem 1 ([2]) *A targeted linear dynamical system (A,I,T) is controllable if and only if its controllability matrix $[C_TB_I, C_TAB_I, C_TA^2B_I, \ldots, C_TA^{n-1}B_I]$ is of full rank.*

The controllability matrix of the targeted linear dynamical system with inputs (A,I,T) is an $l \times mn$ matrix. Thus, being of full rank is equivalent with its rank being equal to l (since $l \leq mn$). Intuitively, this matrix describes all weighted paths from the input nodes to the target nodes in the directed graph associated to the dynamical system. This leads to the notion of "control path" from an input node to a target node and an input node "controlling" a target node. This line of thought can be further developed into a structural formulation of the targeted controllability and into a graph-based solution for it, see[1]. The key results making this possible[3,4] prove that if a system is structurally target controllable, then it is also target controllable in almost all numerical setups of the non-zero entries in A, B and C, except a "thin" set of such matrices.

The problem we focus on and solve in this paper is that of minimizing the set of input nodes needed for the target controllability of a dynamical system. For the linear dynamical system defined by a matrix $A \in \mathbb{R}^{n\times n}$ and a set of target nodes T of size l, the problem is to find the smallest m, $1 \leq m \leq l$, such that for a suitable input set I of size m, the targeted linear dynamical system with inputs (A,I,T) is target controllable.

We add an extra layer of optimization to the target controllability problem, motivated by medicine as our application domain. In medical applications, the input functions mimic the effect of drug delivery, with the input nodes being targets of commercially available drugs. Consequently, we introduce in our mathematical formulation an additional set of so-called preferred nodes $P \subseteq \{1,2,\ldots,n\}$, with the aim of selecting in the input set I as many preferred nodes as possible. The problem in this case becomes the following. For the linear dynamical system defined by a matrix $A \in \mathbb{R}^{n\times n}$, a set of target nodes T and a set of preferred nodes P, the problem is to find a smallest-sized input set I whose intersection with P is maximal, such that the targeted linear dynamical system with inputs (A,I,T) is controllable, i.e., such that matrix $[C_TB_I, C_TAB_I, C_TA^2B_I, \ldots, C_TA^{n-1}B_I]$ is of full rank.

The optimization version of the above-defined target controllability problem has been shown to be NP-hard[5], meaning that an exact solution may require a prohibitive amount of time, exponential in the number of nodes. We focus in this paper on the next most-feasible objective, an efficient heuristic solution that comes, however, with no guarantee of being optimal.

To decide whether the matrix has full rank we use the Math.NET Numerics numerical library[6] and its function "IsFullRank". The algorithm behind this function aims to build a QR decomposition for the Kalman matrix by Householder transformations. The algorithm builds a sequence of matrices Q_k, R_k, $k \geq 1$, all of them with the property that the Kalman matrix is equal to Q_kR_k, Q_k is a unitary matrix, and R_k has its first k columns form an upper triangular matrix. If at any step of this procedure, the Householder transformation fails to extend the non-zero diagonal of the current R matrix, then the algorithm concludes immediately that the matrix does not have full rank, without calculating its rank, thus speeding up the computation. The numerical computations we do repeatedly in our software implementation are parallelized and efficient even for matrices with thousands of rows and columns. For a matrix $m \times n$, with $m \leq n$, the number of operations required in the full calculations for the QR decomposition (in other words, the number of operations required for a full rank matrix) is $2m^2n - (2m^3)/3$.

The Greedy algorithm for structural constrained target controllability

The algorithm was first introduced in[7] and then improved in[1,5]. It uses a graph-based approach to solving the structural controllability problem. Intuitively, the algorithm starts the search from the target nodes and builds the control paths backwards in successive steps and selecting each time the edges locally deemed most promising. When these control paths cannot be further extended and improved, their starting nodes are returned as the input nodes for control. The algorithm is based on the graph-theoretical results of[8]. These results give a necessary conditions for a set of paths to be control paths, by connecting the structural target controllability problem to the linking graph: a set of control paths from the input nodes to the target nodes must satisfy the property that no two paths intersect at the same distance from their endpoints. However, this condition is only necessary for the structural target controllability, but not sufficient. In other words, there are sets of paths satisfying this

condition that are not control paths and hence, their starting nodes are not controlling the target nodes. For this reason, we validate all solutions offered by the greedy algorithm through a check of their Kalman rank condition.

The algorithm takes as input a network given as a directed graph $G = (V,E)$, and a list of target nodes $T \subset V$. It returns a set of input nodes $I \subset V$ which structurally controls the set T, with the objective of minimizing the number of nodes in I. The algorithm begins at step $i = 0$, and defines $I_0 = T$ and $I = \emptyset$. At each successive step i, it constructs a bipartite graph $G_i = (L_i \sqcup R_i, E_i)$ (where $L_i \sqcup R_i$ denotes the disjoint union of the sets L_i and R_i), with $L_i = V$, $R_i = I_{i-1}$, and $E_i = \{(l,r) \mid l \in L_i, r \in R_i, (l,r) \in E\}$. It then identifies a maximum matching in G_i. Each matched edge corresponds to the left-side node directly controlling the right-side node in the initial graph, and elongates the current corresponding control path starting from its right-side node and ending in a target node. The set of matched left-side nodes becomes I_i and their control will be attempted in the following steps, while the unmatched right-side nodes are added to I. It can be seen that at each step i the nodes in $I_i \cup I$ control all of the nodes in T through control paths of length at most i. This way, no two input nodes will be situated at the same distance from their corresponding target nodes, thus satisfying the graph-based condition for control. The algorithm ends either after a predefined number of steps, in the case of the constrained greedy algorithm, or after $|V|$ steps in the case of the unconstrained greedy algorithm. The left-side nodes left unmatched at the end of the last step are added to I and the entire set I is offered as the output of the algorithm.

As discussed in[1], the output of the algorithm may not structurally control the target set and so, it may not be a valid solution to the structural constrained target controllability problem (such a counterexample is provided in[8]). Thus, an additional verification is necessary, to check if the Kalman rank condition is satisfied, i.e., if the controllability matrix $[C_T B_I, C_T A B_I, C_T A^2 B_I, \ldots, C_T A^{n-1} B_I]$ is of full rank.

Several heuristic criteria exist for the choice of a maximum matching in a graph G_i, when multiple options are available. One of them, focused on minimizing the number of input nodes is to select for each iteration's matching, left-side nodes that already exist in I or that have already been matched at any of the previous steps. These nodes are controlled by nodes in the current set I and selecting them in the matching avoids introducing new input nodes. The constrained version of the structural target control problem, in which we have a set P of *preferred* input nodes, can also be addressed in choosing the matching strategy. The problem in this case becomes one of min-max optimization, where $|I|$ should be minimal, but $|P \cap I|$ should be maximal. This can be solved by the introduction of additional heuristics that would maximize the use of nodes in P in each matching.

The genetic algorithm for structural constrained target controllability

We discuss in this section all the details of how the genetic algorithm is designed and implemented.

Chromosome encoding and the fitness function

A chromosome I consists of a vector of l genes $I = [g_1, \ldots, g_l]$, not necessarily of distinct value, where l is the size of the target set T. As discussed before, for all $1 \leq i \leq l$, $g_i \in V$ controls node $t_i \in T$ and so, in particular, it is an ancestor of t_i in graph G. Keeping with our focus on applications in medicine, where paths encode signalling networks, we aim for short paths between the input nodes g_i and the target nodes t_i. The maximum allowed length for such a path is encoded in the parameter $\max_{path}$. Any time we discuss an ancestor g of node t we mean implicitly that the shortest path from g to t is of length at most $\max_{path}$.

A chromosome $I = [g_1, \ldots, g_l]$ is always checked to ensure that the nodes encoded by its genes are able to control the target set T. This is equivalent to the Kalman matrix corresponding to graph G, input set I and target set T having maximum rank l. All populations, throughout all steps of the algorithm, will consist only of such chromosomes.

The fitness score of chromosome I is defined as the complement of the number of distinct nodes encoded by its genes:

$$f(I) = \frac{(l+1) - |\text{supp}\{b_i | b_i \in I\}|}{l} \cdot 100.$$

Thus, $0 < f(I) \leq 100$. Considering that we are interested in control sets of minimum size, the higher the fitness score of a chromosome, the better its encoded solution is.

To generate a random chromosome, we will first initialize each of its l elements g_i, $1 \leq i \leq l$, with its corresponding target node t_i. Then, for a number of randomly selected genes (as many as indicated by the parameter $\max_{rand}$), we will replace gene g_i with a randomly chosen ancestor of t_i (at distance at most $\max_{path}$ from t_i). Each vector of nodes generated in this way is checked for its Kalman condition: if satisfied, the vector encodes a set of nodes controlling the target set T and it is accepted as a valid output. In this way we make sure that every chromosome, throughout the search for the optimal solution, is a viable solution to the controllability problem, thus avoiding the problem of false positives.

Selection

The selection operator is used to choose which chromosomes in the current generation will contribute offsprings to the next generation. The selection of a chromosome depends only on its fitness in relation to the average fitness of the current generation: the better the fitness, the higher the chance it has to be selected. If the current population consists of chromosomes $I_1, \dots, I_n$, then the probability of selecting chromosome I_i, $1 \le i \le n$ is

$$p(I_i) = \frac{f(I_i)}{\sum_{k=1}^{n} f(I_k)}.$$

Obviously, $0 < p(I_i) < 1$ for any $1 \le i \le n$, so all chromosomes have a chance of being selected.

Crossover

The crossover operator is used to produce a new (valid) offspring chromosome from two parent chromosomes. Each of the offspring chromosome's genes will be directly inherited from one of the two parent chromosomes. The actual parent who will contribute a certain gene is randomly chosen based on the number of occurrences of that gene in the two parent chromosomes: the more often it occurs (in other words, the more efficient its control over the target set), the higher the probability it will be selected for the offspring. For a chromosome $I = [g_1, \dots, g_l]$ and a gene g, we define the number of occurrences of g in I to be $\#_I(g) = |\{1 \le i \le l \mid g_i = g\}|$. Also, for two chromosomes I, I', the number of occurrences of g in I, I' is defined to be $\#_{I,I'}(g) = \#_I(g) + \#_{I'}(g)$.

Consider the parent chromosomes to be $I_1 = [g_{11}, g_{12}, \dots g_{1l}]$ and $I_2 = [g_{21}, g_{22}, \dots g_{2l}]$. For all $1 \le i \le l$, gene g_i of the offspring will be either g_{1i} or g_{2i}. If the genes g_{1i} or g_{2i} are either both preferred (i.e., elements of P), or both not preferred, then the probability distribution is defined as:

$$\begin{aligned} p(g_{1i}) &= \frac{\#_{I_1,I_2}(g_{1i})}{\#_{I_1,I_2}(g_{1i}) + \#_{I_1,I_2}(g_{2i})}, \\ p(g_{2i}) &= \frac{\#_{I_1,I_2}(g_{2i})}{\#_{I_1,I_2}(g_{1i}) + \#_{I_1,I_2}(g_{2i})}. \end{aligned} \tag{3}$$

On the other hand, if one of the two parent genes, say g_{1i}, is a preferred node, while the other is not, we reflect our preference for nodes in P in the selection probability as

$$\begin{aligned} p(g_{1i}) &= \frac{2 * \#_{I_1,I_2}(g_{1i})}{2 * \#_{I_1,I_2}(g_{1i}) + \#_{I_1,I_2}(g_{2i})}, \\ p(g_{2i}) &= \frac{\#_{I_1,I_2}(g_{2i})}{2 * \#_{I_1,I_2}(g_{1i}) + \#_{I_1,I_2}(g_{2i})}. \end{aligned} \tag{4}$$

If the set of nodes obtained as a result of the selection above does not satisfy the Kalman condition, then we do not accept it as a valid solution and we discard it, restarting the crossover operator by selecting two new parent chromosomes. In our numerical experiments, relatively few sets of nodes thus selected failed to satisfy the Kalman condition and this step did not become a bottleneck in our algorithm.

We implemented four different crossover strategies, to allow the focus on the list of preferred nodes, if one is given. We describe them below.

- Weighted random parent: This is the default crossover algorithm, presented above, with the selection probabilities described by equation (3).
- Weighted random preferred parent: In this variant of the algorithm, preferred nodes are selected with a higher probability, as indicated in equation (4). If the two parent genes are both preferred, or none is preferred, then the "Weighted random parent" algorithm is used.
- Dominant parent: Between the two parent genes g_{1i} and g_{21}, the one to be selected is that which occurs more often in its chromosome, i.e., the comparison is between $\#_{I_1}(g_{1i})$ and $\#_{I_2}(g_{2i})$. If the genes have the same number of occurrences, then one is selected randomly.
- Dominant preferred parent: Between the two parent genes, the one on the preferred list is selected. If both or none are preferred, then the Dominant parent variant is applied.

Mutation

The mutation operator is used to change the values of a small number of genes in a chromosome. The probability for a gene of a chromosome to be selected for mutation is given by the parameter $0 \leq p_m < 1$. Thus, on average, each newly generated offspring chromosome has a number of $p_m \times l$ mutated genes. Each gene g_i, $1 \leq i \leq l$ represents an ancestor of its corresponding target node t_i, so g_i getting mutated corresponds to replacing it with another ancestor of t_i; the option of getting g_i again is allowed. The new ancestor is randomly chosen based on the number of occurrences of the corresponding gene within the chromosome: the more often it occurs (i.e., the more efficient its control over the target set), the higher the probability it will be selected for the mutation. Genes encoding preferred ancestors have double weight.

If the newly obtained chromosome is not valid according to the Kalman condition, then we repeat the process with the same genes selected for mutation.

We implemented four different mutation algorithms, to enhance the focus on the list of preferred nodes, if one is given. We describe them below.

- Weighted random ancestor: This is the default mutation algorithm, given above, where the new value for the mutated gene is selected from the ancestors with a probability proportional to the number of its occurrences in the chromosome.
- Weighted random preferred ancestor: This is a variant of the default mutation algorithm, in which the preferred genes have twice their weight.
- Random ancestor: In this variant, the new value for the mutated gene is selected among the ancestors with a uniform probability distribution.
- Random preferred ancestor: In this variant, the new value for the mutated gene is selected among the preferred ancestors with a uniform probability distribution. If no preferred ancestor exists, then its selection among the ancestors is random with a uniform probability distribution.

Implementation

The proposed algorithm has been implemented as a cross-platform stand-alone desktop application written in C#/.NET Core and usable within a command-line interface or under a browser-based graphical interface. The source code and the latest release are available at[9].

For each run, the software requires several files: the list of directed edges, the list of target nodes, and the set of parameters (discussed in the main part of the article). In addition, a file containing the list of preferred nodes can also be given as an optional input. Both the command-line and the graphical user interface implementations return the same type of output data, a JSON file containing all the relevant information of the algorithm run, such as details about the input data, the used parameters, the run time, and the control nodes corresponding to each of the identified solutions.

We parallelized the execution of the most used methods, such as chromosome initialization or crossover. All matrix operations use the Math.NET Numerics library[6]. Further details on the required formats, implementation and usage can be found in the GitHub repository[9].

Example of applying the genetic and the greedy algorithms

We demonstrate in this section the workflow of the two algorithms on a small example network. We are interested in obtaining a total of $N = 80$ solutions (of varying sizes) for each algorithm, where a solution consists of a set of input nodes able to control the given target set. The allowed maximum length for a control path is $max_{path} = 5$. For simplicity reasons, the demonstration does not consider any preferred nodes.

The network

The example network is illustrated in Figure S3. The network can be represented by the graph $G = (V, E)$, where $V = \{1, 2, 3, ..., 16\}$, $|V| = 16$, represents the set of nodes and $E = \{(1,4),(1,5),(2,5),(2,6),(3,7),(4,11),(5,4),(5,9),(5,10),(6,8),(7,8),(9,8),(9,15),(10,14),(11,12),(11,13),(12,13),(13,16),(14,5)\}$, $|E| = 19$, represents the set of edges. The corresponding target set is $T = \{8, 10, 11, 13\}$, $|T| = 4$. The adjacency matrix of the graph is $A \in \mathbb{R}^{|V| \times |V|}$ and the output matrix corresponding to the target set is $C \in \mathbb{R}^{|T| \times |V|}$.

Applying the greedy algorithm

The greedy algorithm performs successive heuristic maximum matchings. One possible run of the algorithm on the example network is shown in Figure S4.

At every step i (corresponding to every row on the figure, starting at 0), each matched node can control the corresponding target node through the highlighted control path of length i. For the step 0, this corresponds to each target node being able to

control itself. There are $max_{path} = 5$ steps performed during one run of the algorithm, i.e., the algorithm extends the control paths one edge at a time.

The result of the algorithm is represented by the set of nodes in I after the last step. The validity of this set is checked through the computation of its corresponding input matrix $B \in \mathbb{R}^{|V|\times|I|}$ and the subsequent Kalman matrix $R = [CB \mid CAB \mid CA^2B \mid \ldots \mid CA^5B]$. If the Kalman matrix is of full rank, then the returned set can indeed control the target set and represents a solution to the problem. When the algorithm is applied $N = 80$ times, the output consists of the smallest valid input sets identified throughout these runs.

Applying the genetic algorithm

The genetic algorithm begins with an initialization stage. In this stage, it computes the adjacency matrix A, its successive powers up to max_{path} (i.e., $A, A^2, \ldots, A^5$), and their products with the output matrix C (i.e., $C, CA, CA^2, \ldots, CA^5$). This allows for quicker computations of the Kalman matrix during the next stages. At the same time, during the computation of the powers of the matrix A, the algorithm creates, for each of the target nodes, a set of their ancestors within a distance of at most max_{path}:

$$\begin{array}{rcl} 8 & : & \{8,6,7,9,2,3,5,14,1,10\} \\ 10 & : & \{10,5,1,2,14,10\} \\ 11 & : & \{11,4,5,1,2,14,10\} \\ 13 & : & \{13,11,12,4,1,5,2,14,10\} \end{array}$$

A gene within a chromosome will encode an ancestor of the corresponding target node. A chromosome will then represent an ordered list of ancestors of the target nodes, whose corresponding set can together control the entire target set. This way, the algorithm does not consider the actual path from a control node to a target node, but only that such a path exists (i.e., the control node is an ancestor of the corresponding target node). Thus, for the generation of random chromosomes, an ancestor is selected randomly for each target node, for example:

Target	:	8	10	11	13
Ancestor	:	1	10	1	14
I	:	$\{1,10,14\}$			

Target	:	8	10	11	13
Ancestor	:	2	1	5	10
I	:	$\{1,2,5,10\}$			

For each newly obtained chromosome, at any stage, the algorithm computes its corresponding input matrix $B \in \mathbb{R}^{|V|\times|I|}$ and the subsequent Kalman matrix $R = [CB \mid CAB \mid CA^2B \mid \ldots \mid CA^5B]$, whose rank is then checked. If its Kalman matrix is of full rank, then the chromosome is stored into the population, otherwise it is discarded and its generation process is repeated. The fitness of a valid chromosome is inversely proportional to $|I|$.

The population in each generation will consist of exactly $N = 80$ chromosomes. The initial generation is formed only by random chromosomes, however, each subsequent generation will contain the best chromosomes of the previous generation (i.e., the "elites" with the highest fitness), their offspring, plus several new random chromosomes. The offspring of two chromosomes is obtained through a random weighted selection, for each target node, of one of the corresponding ancestors encoded by the parents.

Target	:	8	10	11	13
Chromosome 1	:	1	10	1	14
Chromosome 2	:	2	1	5	10
Offspring	:	1	1	1	10
I	:	$\{1,10\}$			

The mutation within a chromosome works in a similar way. Once a specific gene was selected for mutation, the ancestor that it encodes is randomly replaced with another ancestor of the corresponding target node.

Target	:	8	10	11	13
Offspring Chromosome	:	1	1	1	10
Mutated Chromosome	:	1	1	1	1
I	:	$\{1\}$			

The process is repeated for the predefined number of generations. The sets of ancestor nodes encoded by the chromosomes with the best fitness in the last generation will then be returned as the final solutions to the problem.

The benchmark networks

The topological analysis of the networks analyzed in this paper, i.e., their centrality measures, are in Figure S1. Also, in Figure S2 there is an illustration of how the fitness of the best solutions of the genetic algorithm evolved on the random networks analyzed in this paper.

References

1. Czeizler, E., Wu, K.-C., Gratie, C., Kanhaiya, K. & Petre, I. Structural target controllability of linear networks. *IEEE/ACM Transactions on Comput. Biol. Bioinforma.* **15**, 1217–1228, DOI: 10.1109/tcbb.2018.2797271 (2018).
2. Kalman, R. E., Ho, Y. C. & Narendra, K. S. Controllability of linear dynamical systems. *Contributions to Differ. Equations* **1**, 189–213 (1963).
3. Lin, C.-T. Structural controllability. *IEEE Transactions on Autom. Control.* **19**, 201–208, DOI: 10.1109/TAC.1974.1100557 (1974).
4. Shields, R. & Pearson, J. Structural controllability of multiinput linear systems. *IEEE Transactions on Autom. Control.* **21**, 203–212, DOI: 10.1109/TAC.1976.1101198 (1976).
5. Kanhaiya, K., Czeizler, E., Gratie, C. & Petre, I. Controlling directed protein interaction networks in cancer. *Sci. Reports* **7**, DOI: 10.1038/s41598-017-10491-y (2017).
6. MathNET. Math.net numerics (2019). Available at https://numerics.mathdotnet.com/.
7. Gao, J., Liu, Y.-Y., D'Souza, R. M. & Barabási, A.-L. Target control of complex networks. *Nat. Commun.* **5**, DOI: 10.1038/ncomms6415 (2014).
8. Murota, K. & Poljak, S. Note on a graph-theoretic criterion for structural output controllability. *IEEE Transactions on Autom. Control.* **35**, 939–942, DOI: 10.1109/9.58507 (1990).
9. Popescu, V. GeneticAlgNetControl (2021). Available at https://github.com/Vilksar/GeneticAlgNetControl, version 1.0.

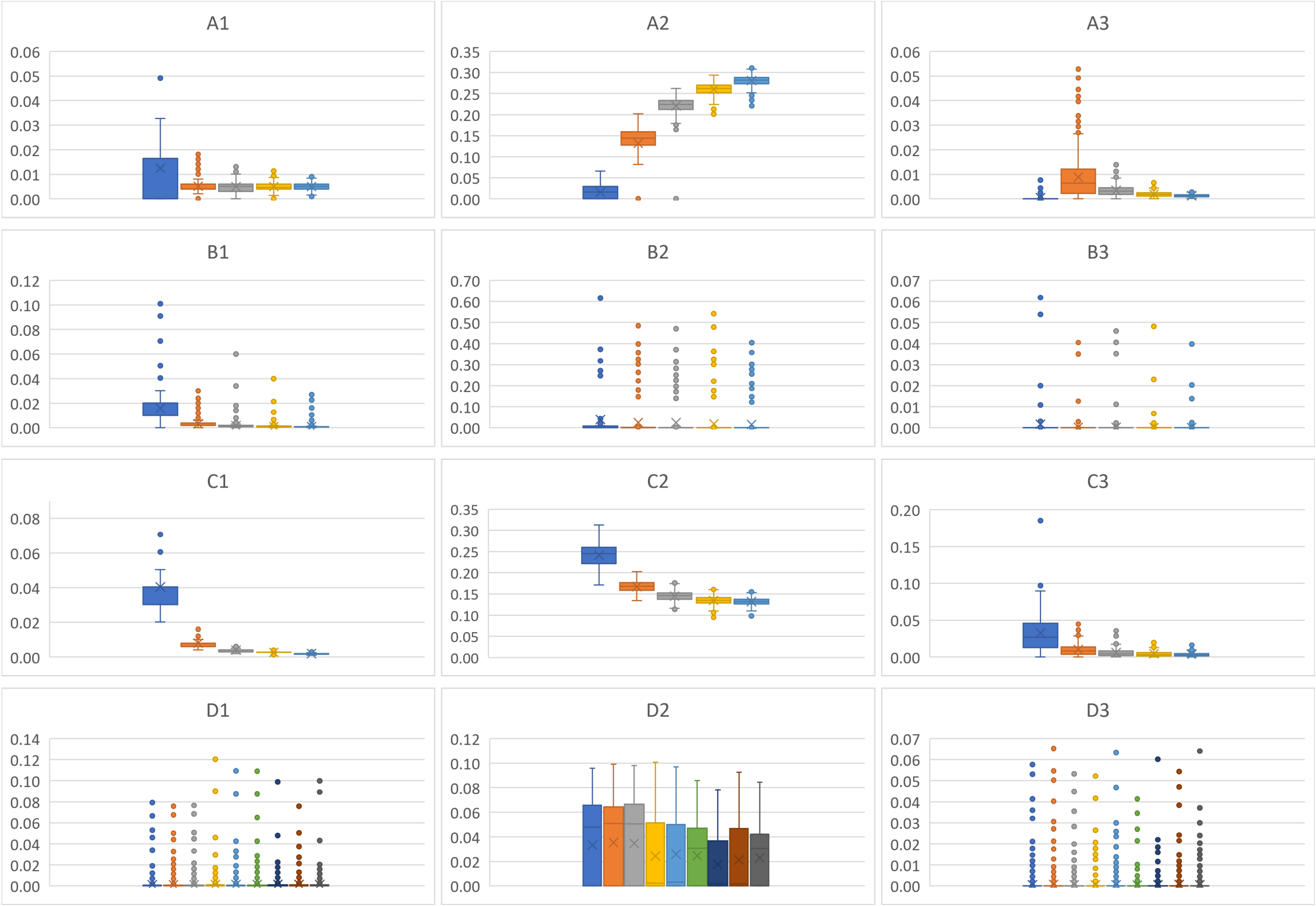

Figure S1. The centrality measures of the analyzed networks. **A**: Erdős-Rényi networks, **B**: Scale-Free networks, **C**: Small World networks, **D**: biological networks; **column 1**: out-degree, **column 2**: closeness degree, **column 3**: betweenness degree; **from left to right, A-C**: the networks with 100/500/1000/1500/2000 nodes, **from left to right, D**: Breast DEF, Breast HCC1428, Breast MBA-MB-361, Ovarian DEF, Ovarian O1946, Ovarian OVCA8, Pancreatic AsPC-1, Pancreatic DEF, and Pancreatic KP-3 cancer networks. The boxes indicate the interquartile range (IQR) Q1-Q3, and the whiskers extend to the furthest point within 1.5×IQR. The points beyond the whiskers are shown with dots and considered to be outliers. With a cross we indicate the mean values of the data points.

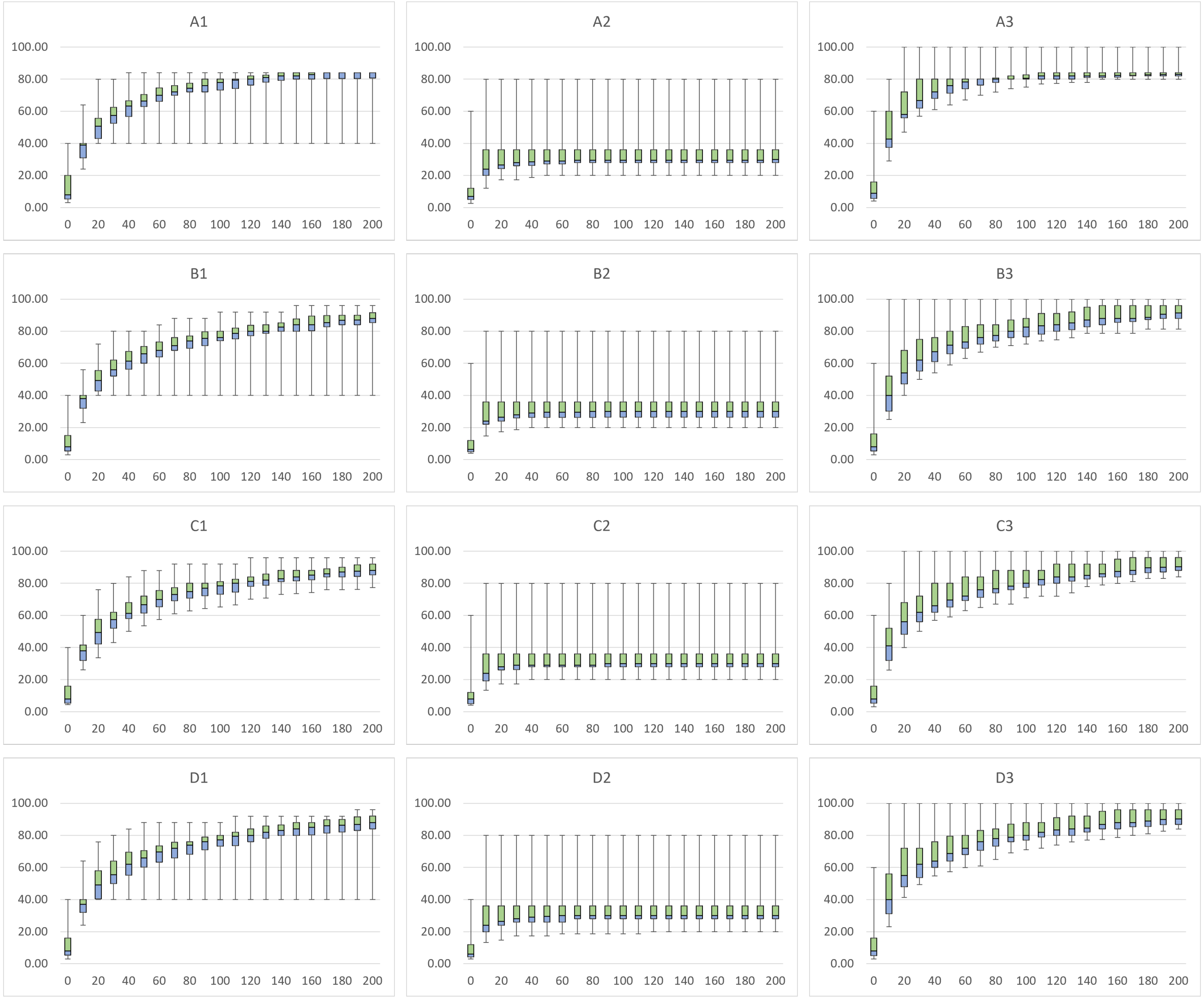

Figure S2. The fitness of the best chromosomes over the first 200 generations of the genetic algorithm for the random networks. **A**: maximum control path of 5, **B**: maximum control path of 15, **C**: maximum control path of 30, **D**: maximum control path of 50. **column 1**: Erdős-Rényi networks, **column 2**: Scale-Free networks, **column 3**: Small World networks.

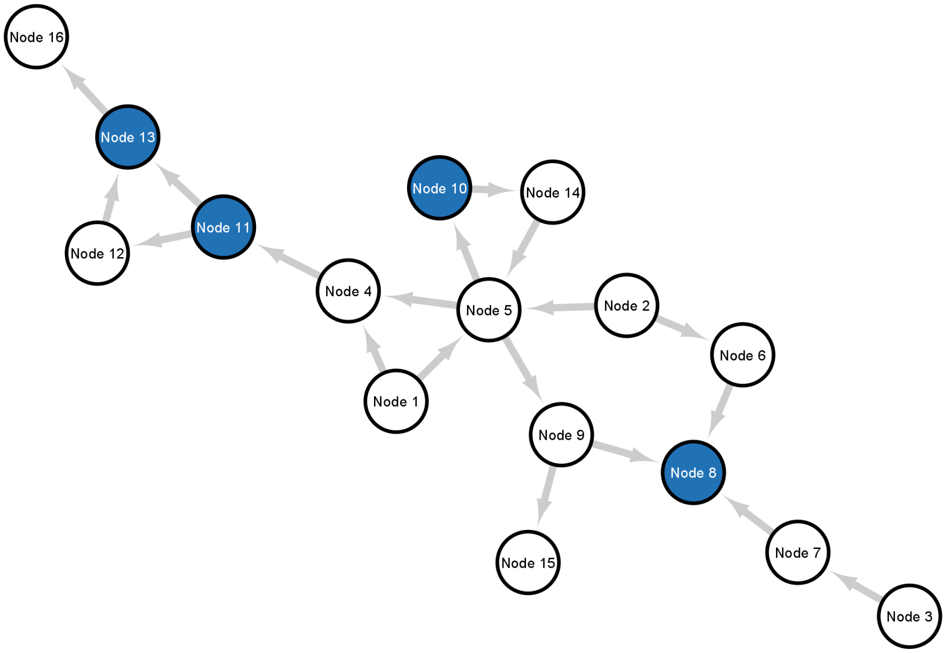

Figure S3. The example network for illustrating the algorithms. The network contains 16 nodes, out of which 4 target nodes, and 19 edges. **in black**: target nodes.

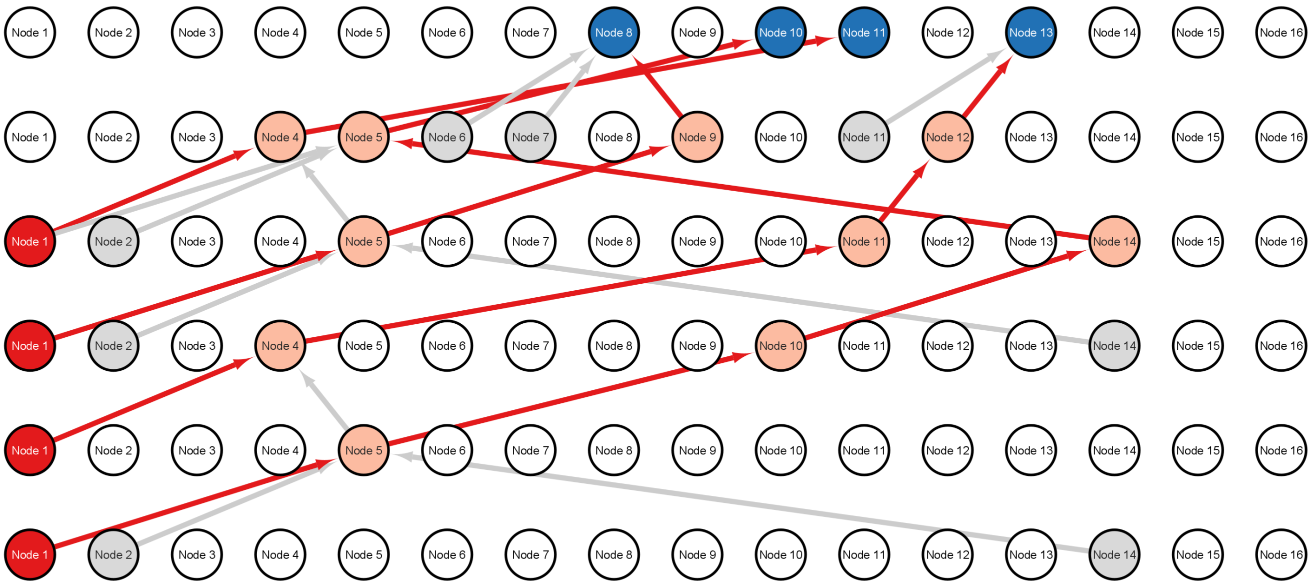

Figure S4. The successive maximum matchings performed by the greedy algorithm. The figure shows only one possibility of choosing the matched edges at each step, among multiple possible. **in black**: target nodes, **in light red**: intermediary matched nodes, **in light gray**: intermediary unmatched nodes, **in dark red**: input nodes.

Table S1. The supplementary tables.

Table	Description
Supplementary Table 1 (Overview of the analyzed networks)	An overview of the analyzed networks, containing the source of the network and associated target set(s), the number of nodes, edges, and the average node degree, as well as the target nodes and preferred nodes (where applicable), both in absolute value and as a percentage of the total number of nodes in the network. Additionally, we included several centrality measure values (out-degree, closeness degree, and betweenness degree) for each node in all of the networks.
Supplementary Table 2 (Results of the three algorithms)	Statistics of the results of the three algorithms (genetic, constrained greedy, and unconstrained greedy) over multiple iterations for each network, using different analysis setups (without preferred nodes, with preferred nodes, and with preferred nodes and edge-subsampling).
Supplementary Table 3 (Therapeutically relevant results of the three algorithms)	The therapeutically relevant results of the three algorithms (genetic, constrained greedy, and unconstrained greedy) over multiple iterations for each biological network, using different setups (with preferred nodes, and with preferred nodes and edge-subsampling). For each protein and each network, we counted the number of independent algorithm iterations in which it was identified as a preferred control input.
